# Right ventricular metastasis from unclassified Renal Cell Carcinoma

**DOI:** 10.22088/cjim.10.2.235

**Published:** 2019

**Authors:** Oluwaseun A. Akinseye, Devarshi R. Ardeshna, Meron K. Teshome, Shadwan Alsafwah

**Affiliations:** 1 Division of Cardiovascular Diseases, Department of Medicine, University of Tennessee Health Science Center, Memphis, Tennessee, USA; 2 Department of Medicine, University of Tennessee College of Medicine, Memphis, Tennessee, USA

**Keywords:** Cardiac tumor, Metastasis, Right ventricular mass, unclassified renal cell carcinoma

## Abstract

**Background:**

Cardiac metastasis of unclassified renal cell carcinoma (RCC) subtype is very rare, and even more so is an isolated right ventricular (RV) metastasis without vena cava extension or right atrial involvement. To the best of our knowledge, this is the first report of a cardiac metastasis of an unclassified RCC (an aggressive RCC) without vena cava extension.

**Case Presentation:**

A 61-year-old African American male with past medical history of hypertension and schizophrenia presented to the emergency room following 2 episodes of syncope and 3-month history of progressive neck mass. CT scan of neck, abdomen and pelvis showed bulky left cervical, supraclavicular and axillary lymph node, mass in anterior aspect of heart, and multiple solid left renal masses and probable right renal mass. Echocardiogram revealed a large RV mass with deformation of the RV free wall suggesting malignant growth. Core biopsy of the right superficial gluteal mass revealed a metastatic poorly differentiated carcinoma of likely renal origin, with a possibility of an unclassified RCC. Due to the extent and burden of metastasis, patient and family members agreed to conservative management and evaluation for hospice care.

**Conclusion:**

Cardiac metastasis of unclassified RCC is rare, and even more so is an isolated RV metastasis without vena cava extension or right atrial involvement, and the present case, to the best of knowledge is the first of such rare presentation.

Metastatic cardiac tumors are more prevalent than primary cardiac tumors (1), and they arise most often from malignant melanoma, lung cancers, breast cancers, esophageal cancers, leukemia and lymphomas (2). Renal cell carcinoma (RCC) metastasis to the heart is rare, even more so is the metastasis of an undifferentiated RCC to the right ventricle (RV) without an inferior vena cava (IVC) or right atrial (RA) extension. The most common mechanism of cardiac metastasis in RCC is through direct tumor thrombus extension through the renal veins and IVC to the right side of the heart (3), and dissemination through hematogenous or lymphatic spread is reported as the second most common mechanism of metastasis (4). The clear cell RCC is the most commonly reported histopathology to the heart, and to the best of our knowledge, this is the first reported case of an unclassified RCC metastasis to the heart. We report a metastatic unclassified and poor differentiated RCC to the RV without a contiguous IVC or RA involvement. 

## Case Presentation

A 61-year-old African American male with past medical history of hypertension and schizophrenia presented to the emergency room following 2 episodes of syncope. 

**Figure 1:**
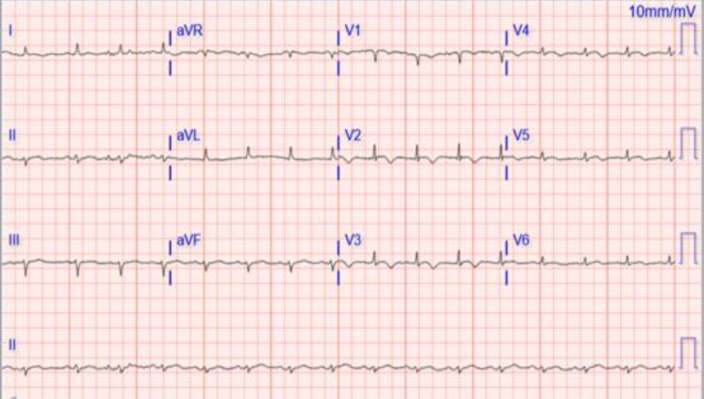
Electrocardiogram showing normal sinus rhythm, left axis deviation, low voltage and mild t-wave inversion in V2 – V4

He reported 3 month history of progressive neck mass. Physical examination revealed a temperature of 37.7 degrees, blood pressure of 130/87 mmHg, pulse of 92 bpm, and respiratory rate of 17 bpm. There was extremely large left sided neck mass extending into the left axilla and multiple palpable left and right cervical lymph nodes. The lungs were clear to auscultation, and there was a 2/6 systolic ejection murmur heard best at the bases. The abdomen was soft and nontender, without palpable organomegaly. There was a 5x2 cm right gluteal non-tender, non-mobile mass with central ulceration and also a 2x2 cm spherical mass at the left upper back with small central ulceration. There was 2+ pitting edema of the lower extremities bilaterally. There was differential swelling of left upper extremity. Electrocardiogram showed normal sinus rhythm, left axis deviation, low voltage and mild t wave inversion in V2–V4 (figure 1). 

**Figure 2:**
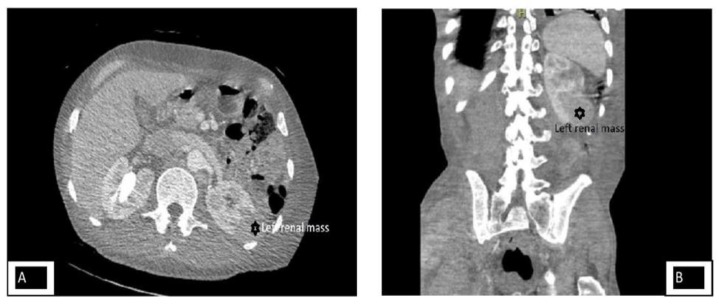
Panel A: Cross-section of CT abdomen and pelvis showing left renal mass. Panel B: Coronal section of CT abdomen and pelvis showing left renal mass

Initial CT scan of the abdomen and pelvis showed diffuse metastatic disease of the visualized lower chest, abdomen and pelvis including superficial soft tissues, left kidney, and probable right kidney. There was diffuse confluent adenopathy and massive right inguinal lymphadenopathy, along with pronounced diffuse anasarca. There are multiple renal masses. There are solid masses arising off of the lateral aspect of the left kidney involving the upper, middle and lower lobe (figure 2). The left kidney mass was described as an exophytic lesion. There was a mass along the anterior aspect of the heart that measures approximately 5 cm but incompletely visualized. CT head was negative. 

**Figure 3:**
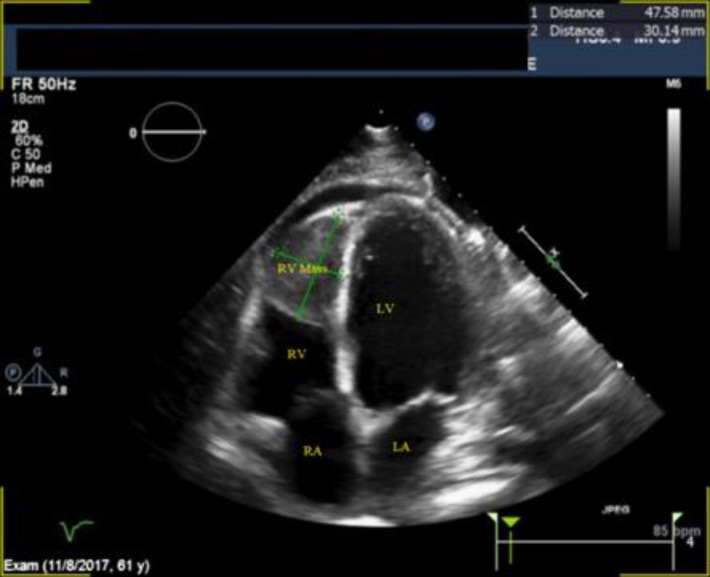
Apical 4-chamber view demonstrating a large mass measuring 4.8 cm x 3.0 cm extending from the apex to the mid right ventricular cavity. LA Left atrium; LV Left ventricle; RA Right atrium; RV Right ventricle

**Figure 4:**
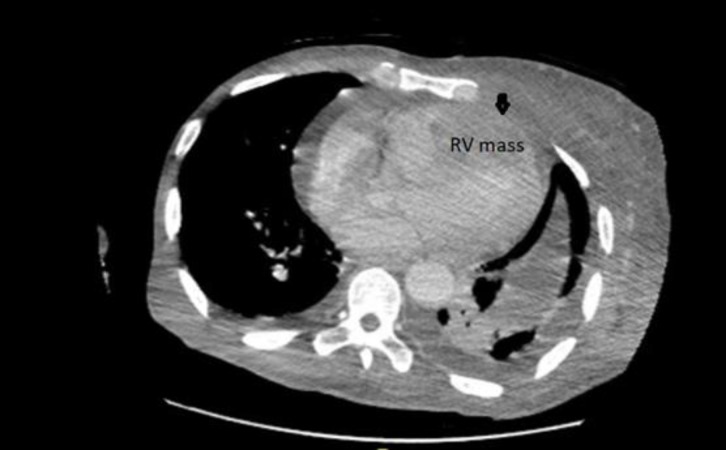
Cross sectional view of CT thorax showing a large right ventricular mass. Arrow: Deformation of right ventricular free wall suggesting infiltration of right ventricular mass into the right ventricular wall myocardium

Transthoracic echocardiogram revealed a large mass measuring 4.8 cm x 3.0 cm extending from the apex to the mid RV cavity, and extending into the RV outflow tract stopping just short of the pulmonic valve (figure 3). There was deformation of the RV free wall suggesting invasion of the myocardial wall and a malignant growth. There was mild RV enlargement, with normal function of the segments not involved in mass. There RA was mildly dilated with no mass seen, and there was no evidence of thrombus in the IVC. There was a small pericardial effusion without echocardiographic evidence of tamponade. A dedicated CT of the thorax revealed a mildly enlarged heart, and a filling defect in the RV measuring 6 cm extending to the apex and suspicious for malignancy (figure 4). 

**Figure 5:**
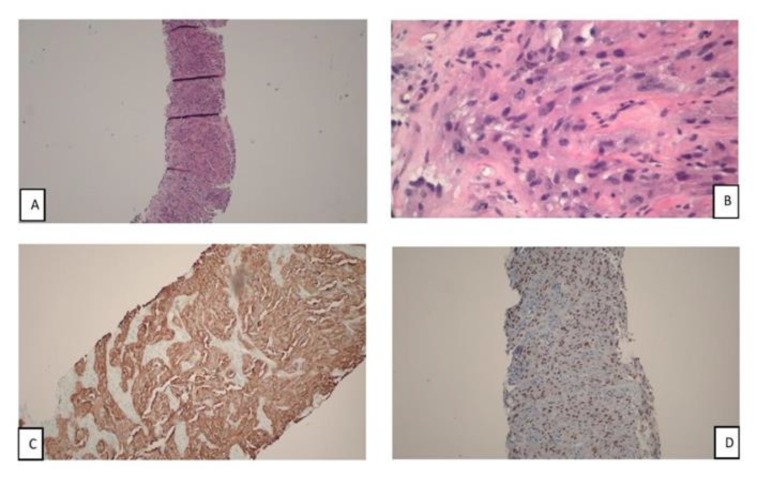
Pathology of right gluteal mass biopsy suggesting metastatic carcinoma of renal origin, possibly of an unclassified renal cell carcinoma. Panel A & B showing magnification x 4 and magnification x 40 respectively. Tumor cells are arranged in solid nests having abundant eosinophilic cytoplasm, central nucleus and conspicuous nucleoli. Panel C: Pancytokeratin expression in tumor cells. Panel D: PAX8 expression in tumor cells with strong nuclear staining

A core biopsy of the right superficial gluteal mass revealed a tumor arranged in solid nests having abundant eosinophilic cytoplasm, central nucleus and conspicuous nucleoli, with areas of coagulative tumor necrosis, and individual tumor cell exhibiting high-grade atypia with pleomorphism (figure 5). 

On immunohistochemistry, the tumor cells expressed pacytokeratin, PAX 8, vimentin and CD 10 (weakly). The tumor cells are negative for S 100, Melan-A and RCC. Immunostains for CD 117 and CK 7 were performed, however were uninterpretable due to tissue depletion. Morphology and immunohistochemical profile favor a metastatic poorly differentiated carcinoma of likely renal origin, with a possibility of an unclassified RCC. Oncology was consulted and they suggested a diffuse metastatic malignancy of primary renal origin with poor prognosis. Oncology advised that the patient should follow up in outpatient cancer clinic. Due to extent and burden of metastasis, patient and family members agreed to conservative management and patient was placed in hospice and comfort care with no further aggressive management. He was discharged from the hospital to hospice and to follow-up with the cancer clinic, although there were no records that he kept the appointments and he was lost to follow-up.

## Discussion

RCC metastasis to the heart is rare. Most secondary tumor metastasis to the heart often arises from malignant melanoma, lung cancers, breast cancers, esophageal cancers, leukemia and lymphomas (2). RCC presents as a localized or locally advanced tumor in approximately 45% and 25% of patients, respectively, and 30% of patients may have metastasis at the time of diagnosis (5). The most common site for RCC metastasis is lungs, bone, soft tissues, liver and central nervous system (5). With cardiac metastasis, a direct tumor extension through the renal veins and IVC to the right side of the heart is the most common mechanism of spread, followed by dissemination through hematogenous or lymphatic spread (3, 4, 6). The histopathology of most reported cardiac metastatic RCC is the clear cell subtype. Unclassified RCC subtype is a very rare variant and accounts for approximately 3-5% of RCC cases (7). 

They possess adverse histopathological features and aggressive biological potential; are usually advanced at the time of diagnosis, and associated with poor clinical outcome and higher mortality compared to other RCC subtypes (8, 9). Our patient presented with syncope and was found to have diffuse metastatic disease of the lower chest, abdomen and pelvis including superficial soft tissues, diffuse confluent lymphadenopathies and an RV mass, with a core biopsy of gluteal mass showing metastatic poorly differentiated carcinoma of likely renal origin, with a possibility of an unclassified RCC. To the best of our knowledge, this is the first reported case of a poorly differentiated unclassified RCC metastasis to the RV, and without contiguous IVC or RA involvement. There was also invasion of the RV myocardial free wall. The episode of syncope may due to intermittent obstruction of flow through the RV outflow tract with subsequent reduction in LV preload and forward flow, although at the time of echocardiography, there was no flow limitation or RV outflow obstruction. The possibility of tachydysrhythmias and conduction defects has a cause of syncope in patients with cardiac metastatic RCC has also been reported likely due to re-entry around the border with the myocardium, accessory bundles resulting in ventricular pre-excitation and valvular interference of the tumor mass (4, 5). Metastatic RCC, unlike other neoplasm are highly resistant to chemotherapeutic, hormonal and radiation therapy, however recent targeted therapeutic agents such as (sunitinib, sorafenib, pazopanib, axitinib, bevacizumab, temsirolimus and everolimus) are now standard therapy and these tumor cells have shown sensitivity to immunotherapy such as interleukin 2 and interferon (10, 11). Surgical resection may be considered for isolated intracavitary cardiac metastasis and appears to have a good prognosis, however most presentations are late with diffuse metastasis and treatment is palliative (10, 11). A combination of both surgical resection in isolated cardiac metastatic cases or metastasectomy if feasible and novel chemotherapy agents may produce a desired result of both palliation and cure (10, 12). 

In conclusion, RCC metastasis to the heart is rare, and it commonly arises as a direct tumor thrombus extension into the RA through the IVC especially in clear cell RCC or from either hematogenous or lymphatic spread. Cardiac metastasis of unclassified RCC is rare, and even more so is an isolated RV metastasis without IVC or RA involvement, and the present case is one of such rare presentation. 

## Conflict of Interest:

None declared.
